# THE CANNABIS AND OLDER PERSONS STUDY: CAN WE AT LEAST DEFINE IT AS A FORM OF COMPLEMENTARY AND ALTERNATIVE MEDICINE?

**DOI:** 10.1093/geroni/igae098.1240

**Published:** 2024-12-31

**Authors:** Brian Kaskie

**Affiliations:** Chair

## Abstract

The Cannabis and Older Persons Study has examined the increasing use of cannabis among older persons since 2016. This year’s COPS symposium features panelists trained in community health, gerontology, medicine, psychology and public health, each offering latest research findings and considering cannabis as a form of complementary and alternative medicine. Annie Nguyen and her colleagues from the University of California, San Diego present a qualitative analysis of interviews completed with physicians and other clinicians affiliated with a geriatric medical clinic, focusing on their perspectives about cannabis as a form of complementary and alternative medicine. Angela Bryan and her team from the University of Colorado, Boulder identify more than 200 older persons who completed the “Cannabis and Your Health” survey and compare those who use cannabis for purposes of self-care and contrasts outcomes related to balance, mood and memory with those who do not use cannabis. Julie Bobitt from University of Illinois Chicago uses a socio-ecological framework to identify multi-level factors associated with cannabis use among 30 older women with HIV. Fadi Martinos examines more than 12,000 persons over 50 who completed the California Health Interview Survey, and compares cannabis use among those who identify as informal care givers and those who do not. Together, these studies reflect how scientific understanding of cannabis use among older persons has been informed by varied disciplinary perspectives, and whether researchers consider cannabis use among older adults as a complementary and alternative form of medicine.

